# Ceftazidime-Avibactam Therapy Versus Ceftazidime-Avibactam-Based Combination Therapy in Patients With Carbapenem-Resistant Gram-Negative Pathogens: A Meta-Analysis

**DOI:** 10.3389/fphar.2021.707499

**Published:** 2021-09-14

**Authors:** Dan Li, Fan Fei, Hua Yu, Xiangning Huang, Shanshan Long, Hao Zhou, Jie Zhang

**Affiliations:** ^1^ School of Medicine, University of Electronic Science and Technology of China, Chengdu, China; ^2^ Department of Laboratory Medicine, Sichuan Provincial People’s Hospital, University of Electronic Science and Technology of China, Chengdu, China; ^3^ Department of Laboratory Medicine, Medical Center Hospital of Qionglai City, Chengdu, China; ^4^ Department of Neurosurgery, Sichuan Provincial People’s Hospital, University of Electronic Science and Technology of China, Chengdu, China; ^5^ Department of Stomotology, Sichuan Provincial People’s Hospital, University of Electronic Science and Technology of China, Chengdu, China

**Keywords:** ceftazidime-avibactam therapy, combination therapy, carbapenem-resistant gram-negative pathogen, efficacy, meta-analysis

## Abstract

**Objective:** To systematically review and compare the efficacy and posttreatment resistance of ceftazidime-avibactam therapy and ceftazidime-avibactam-based combination therapy in patients with Gram-negative pathogens.

**Methods:** PubMed, Embase, Web of Science, CNKI, and Wanfang Data databases were searched from their inception up to March 31, 2021, to obtain studies on ceftazidime-avibactam therapy versus ceftazidime-avibactam-based combination therapy in patients with carbapenem-resistant Gram-negative pathogens. The primary outcome was mortality rate, and the second outcomes were microbiologically negative, clinical success, and the development of resistance after ceftazidime-avibactam treatment.

**Results:** Seventeen studies representing 1,435 patients (837 received ceftazidime-avibactam-based combination therapy and 598 received ceftazidime-avibactam therapy) were included in the meta-analysis. The results of the meta-analysis showed that no statistically significant difference was found on mortality rate (Petos odds ratio (OR) = 1.03, 95% confidence interval (CI) 0.79–1.34), microbiologically negative (OR = 0.99, 95% CI 0.54–1.81), and clinical success (OR =0.95, 95% CI 0.64–1.39) between ceftazidime-avibactam-based combination therapy and ceftazidime-avibactam therapy. Although there was no difference in posttreatment resistance of ceftazidime-avibactam (OR = 0.65, 95% CI 0.34–1.26) in all included studies, a trend favoring the combination therapy was found (according to the pooled three studies, OR = 0.18, 95% CI 0.04–0.78).

**Conclusions:** The current evidence suggests that ceftazidime-avibactam-based combination therapy may not have beneficial effects on mortality, microbiologically negative, and clinical success to patients with carbapenem-resistant Gram-negative pathogens. A trend of posttreatment resistance occurred more likely in ceftazidime-avibactam therapy than the combination therapy. Due to the limited number of studies that can be included, additional high-quality studies are needed to verify the above conclusions.

## Introduction

Infections caused by carbapenem-resistant Gram-negative bacilli (CRGN) are challenging and associated with a high mortality rate (CDC, 2019); it spreads rapidly in the past decade causing a huge public health burden (Bassetti et al., 2017; Avendano et al., 2020; Zhen et al., 2021). Until now, several antimicrobial agents have been developed to fight against these superbugs, among which, ceftazidime-avibactam (CAZ-AVI) is a novel antibiotic combination, consisting of a third-generation cephalosporin (ceftazidime) and a synthetic β-lactamase inhibitor (avibactam). The special chemical structure allows avibactam to bond closely to and inactivate class A, class C, and some class D β-lactamases, but not class B metallo-β-lactamases (Zhanel et al., 2013).

Previous *in vitro* studies (Gaibani et al., 2017; Romanelli et al., 2020) demonstrated that the CAZ-AVI activity was enhanced after combining other agents. However, the results of combination therapy are inconclusive in real-world experiences (Tumbarello et al., 2015; Sousa et al., 2018). Additionally, phase 3 randomized clinical programs have assessed the efficacy of CAZ-AVI compared with other therapies (Carmeli et al., 2016; Wagenlehner et al., 2016) but not with CAZ-AVI-based combination therapy. In 2015, CAZ-AVI was first approved for marketing by the US Food and Drug Administration (FDA) for the treatment of adults (age >18) with serious infections including complicated intra-abdominal infection (cIAI) (along with metronidazole) and complicated urinary tract infection (cUTI) (Administration USFaD., 2015). This scenario occurred only when there were limited or no other therapeutic options. Subsequently, it was approved by the European Medicines Agency (EMA) in 2016 (Shirley, 2018), by the National Medical Products Administration in China in 2019 (14) and in other countries (15, 16). A wealth of CAZ-AVI-treating CRGN studies in real-world practice has been reported. There has been a dispute on whether monotherapy therapy would be inferior to combination therapy (15). Also, this debate occurred in the posttreatment CAZ-AVI scenario (Shields et al., 2018; Tumbarello et al., 2019; Ackley et al., 2020; Castón et al., 2020; Iannaccone et al., 2020; Tumbarello et al., 2021).

Therefore, a meta-analysis aiming to systematically compare the mortality rate, microbiologically negative, clinical success, and the development of resistance between CAZ-AVI therapy and CAZ-AVI-based combination therapy was conducted.

## Methods

### Reporting Guideline

The meta-analysis was reported according to the Preferred Reporting Items for Systematic reviews and Meta-Analyses (PRISMA) guidelines (Page et al., 2021). The PRISMA checklist was provided as Supplementary Material.

### Database Search

Multiple databases, including PubMed, Embase, Web of Science, CNKI, and Wanfang Data, were searched for the studies on CAZ-AVI therapy and CAZ-AVI-based combination therapy for the treatment of infections caused by CRGN (up to March 31, 2021). The search keywords were “ceftazidime avibactam” and “carbapenem resistant. The search strategy was provided as Supplementary Material. The references of relevant articles (both original research and review) were also inspected. The article search and reference inspection were performed by two researchers independently screening the titles and abstracts and examining the full texts and reference lists according to the inclusion and exclusion criteria listed below and (Bassetti et al., 2017) cross-checking each other’s studies.

Inclusion criteria:

i) All types of clinical studies were included: both randomized controlled trials (RCTs) and observational studies (retrospective and prospective).
ii) Studies with availability separate data on CAZ-AVI therapy and any CAZ-AVI-based combination therapy.
iii) Studies reported more than ten adult patients (age >18) with infections caused by CRGN.
iv) No language restrictions on the articles: eligible studies published in any languages were all included.

Exclusion criteria:

i) *In vitro* studies, reviews, or case reports (*n* < 10).
ii) Data on CAZ-AVI therapy or CAZ-AVI-based combination regimen were not reported.

In case of insufficient data provided in the eligible articles, corresponding authors were contacted for more information.

### Data Extraction

The primary outcome was the mortality rate at an appropriate time point (14, 30, and 90 days, in hospital), and the secondary outcomes were microbiologically negative, clinical success, and the development of resistance after CAZ-AVI treatments. Two researchers independently extracted and cross-checked the studies to ensure the selected studies were unique based on the following information of the studies: the first author, published year, country, study design, patient demographics (age, sex, and number of patients), bacteria, carbapenemase, the severity of illness, comorbidity index, combination agents, and infection type.

### Quality Assessment and Definitions

The Risk of Bias in Nonrandomized Studies of Interventions (ROBINS-I) tool (Sterne et al., 2016a) was used to evaluate the risk of bias in observational studies (11 cohort studies and 1 case-cohort study). The tool includes seven bias domains: confounding domain, domain of selection of participants, classification domain, deviations domain, missing data, measurement domain, and domain of selection of the reported result. The risk of bias for individual studies was accessed by two researchers answering signal questions according to a hypothetical randomized trial. According to the design of the study, the Agency for Healthcare Research and Quality (AHRQ) tool (Rostom et al., 2004) was applied to evaluate the residual five studies (two case series and three cross-sectional studies). Each study was assessed independently by two researchers, and if there was a dispute, a third researcher was employed to resolve the dispute.

The definitions were based on studies included in this meta-analysis. Combination therapy was defined as one or more agents combined with CAZ-AVI for at least 24 h of treatment. Clinical success was defined as the resolution or remarkable improvement of the symptoms and signs of infection caused by CRGN. Microbiologically negative was defined as negative culture from blood or specific sites at the end of the treatment. CRGN was defined as resistance to any carbapenem using an automated or manual methodology.

### Statistical Analysis

RevMan 5.3 software was used to perform the meta-analysis. Pooled odds ratios (ORs) or Peto ORs and 95% confidence intervals (CIs) were computed in the analysis. *I* ^2^ and Q statistics were used to evaluate the heterogeneity. *I* ^2^ > 50% and *p* < 0.1 are assumed as clinical heterogeneity. A fixed-effects model was performed for the meta-analysis. Subgroup analyses were performed according to the endpoint of mortality. Sensitivity analyses were performed as well. *p*-value less than 0.05 was considered significant.

## Results

### Study Search and Characteristics

**FIGURE 1:**
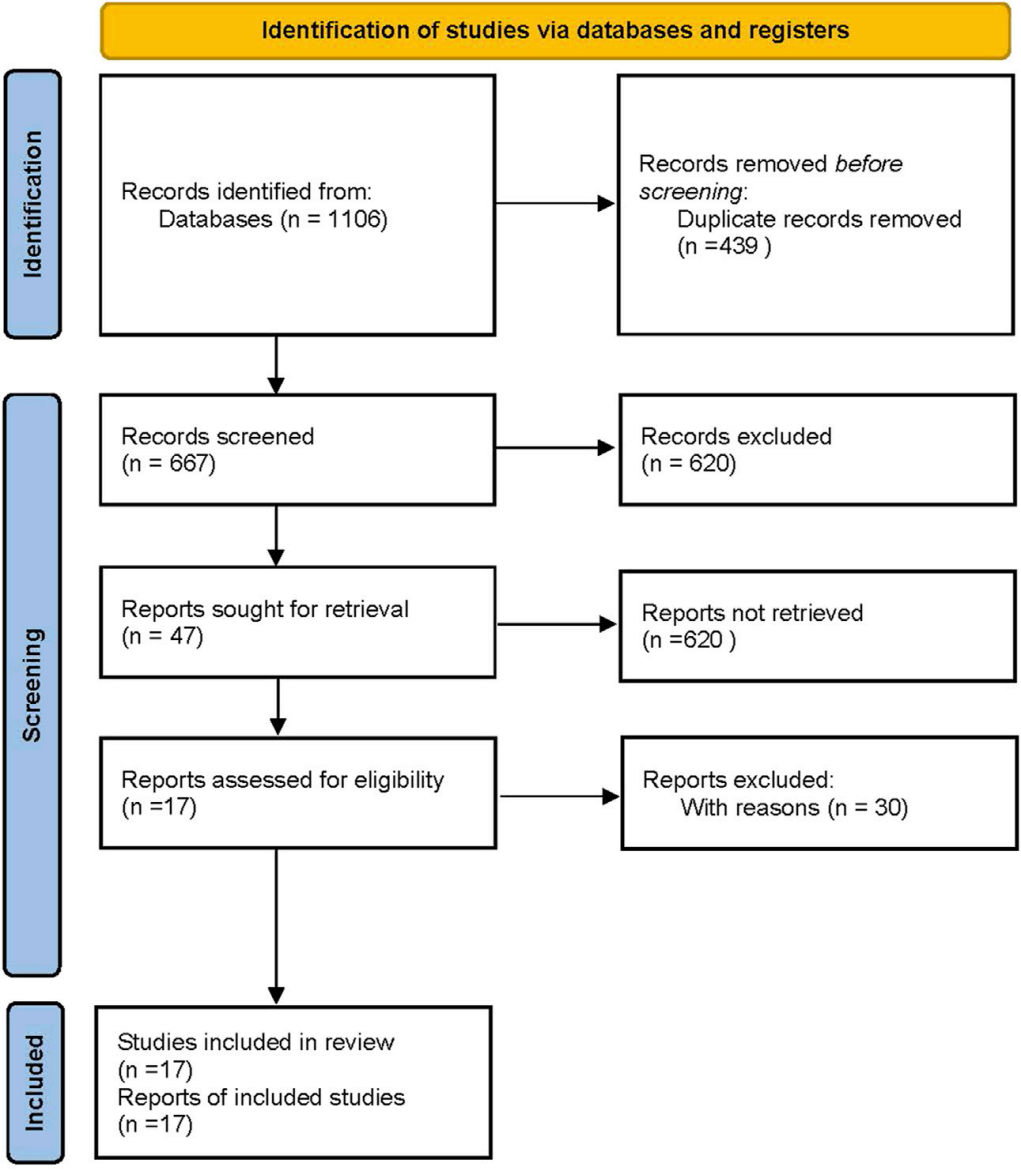
Flow diagram of meta-analysis generated by PRISMA 2020 (Haddaway et al., 2021).

The database search described in Database Search generated 17 eligible articles (Sousa et al., 2018; Kuang et al., 2020; Guimarães et al., 2019; Rathish et al., 2021; Shields et al., 2018; Tumbarello et al., 2019; Ackley et al., 2020; Iannaccone et al., 2020; Castón et al., 2020; Tumbarello et al., 2021; Zhu et al., 2021; King et al., 2017; Temkin et al., 2017; De la Calle et al., 2019; Chen et al., 2020; Jiang et al., 2020; Jorgensen et al., 2020). Specifically, 1,106 references were found by searching for the keywords: “ceftazidime avibactam” and “carbapenem resistant.” After removing the duplicates, 667 potentially relevant articles were left. The 620 records containing only titles and abstracts were then excluded. The remaining 47 studies were further screened using the inclusion and exclusion criteria and returned 17 articles. The details of the database search flow are shown in the study selection flowchart (Figure 1). We used the PRISMA2020 tool (R package and ShinyApp for making PRISMA2020 flow diagrams) (Haddaway et al., 2021) to generate the flow diagram (Figure 1).

The basic information of the 17 articles included in the meta-analysis was summarized (Table 1). There are 11 cohort studies, one case-cohort study, two case series, and three case-sectional studies and all of them were retrospective observational. In total, 1,435 patients were included in this analysis, and the number of patients for different studies ranged from 10 (Chen et al., 2020) to 577 (Tumbarello et al., 2021). The studies were published between 2017 and 2021. Four studies were from the USA (King et al., 2017; Shields et al., 2018; Ackley et al., 2020; Jorgensen et al., 2020), three were from Spain (Sousa et al., 2018; De la Calle et al., 2019; Castón et al., 2020), four were from China (Chen et al., 2020; Jiang et al., 2020; Kuang et al., 2020; Zhu et al., 2021), three were from Italy (Tumbarello et al., 2019; Iannaccone et al., 2020; Tumbarello et al., 2021), one was from Brazil (Guimarães et al., 2019), one was from India (Rathish et al., 2021), and one was from Europe and Australia (Temkin et al., 2017). In terms of the pathogens, most of the studies reported carbapenem-resistant Enterobacteriaceae (CRE) with carbapenem-resistant *Klebsiella pneumoniae* (CKP) as the most frequent, while one study included both CKP and carbapenem-resistant *Pseudomonas aeruginosa* (CRPA) (Temkin et al., 2017) and one study reported CRE, CRPA, and *Acinetobacter baumannii* (AB) (Kuang et al., 2020). As for infection types, most studies demonstrated more than one type of infection, while two were only bloodstream infections (BSI) (Tumbarello et al., 2019; Iannaccone et al., 2020), and one was pneumonia (Jiang et al., 2020). Reagents that have been combined with CAZ-AVI therapy include aminoglycosides (AMG), polymyxin (PM), tigecycline (TGC), colistin (COL), amikacin (AMK), imipenem (IMP), gentamicin (GEN), ciprofloxacin (CIP), meropenem (MEM), fosfomycin (FOS), carbapenems (CAP), fluoroquinolone (FLU), minocycline (MIN), and sulfamethoxazole-trimethoprim (SUL-TRI). Patients were treated by CAZ-AVI and observed for at least 72 h in 12 studies (Temkin et al., 2017; Shields et al., 2018; De la Calle et al., 2019; Tumbarello et al., 2019; Ackley et al., 2020; Castón et al., 2020; Chen et al., 2020; Jiang et al., 2020; Jorgensen et al., 2020; Kuang et al., 2020; Tumbarello et al., 2021; Zhu et al., 2021), for at least 48 h in three studies (Sousa et al., 2018; Guimarães et al., 2019; Rathish et al., 2021), and for at least 24 h in one study (King et al., 2017). There is only one study in which the treatment time was not reported (Iannaccone et al., 2020).

**TABLE 1:** Characteristics of the 17 studies included in the meta-analysis.

Study King et al. (2017); Temkin et al. (2017); Shields et al. (2018); Sousa et al. (2018); De la Calle et al. (2019); Guimarães et al. (2019); Tumbarello et al. (2019); Ackley et al. (2020); Castón et al. (2020); Chen et al. (2020); Iannaccone et al. (2020); Jiang et al. (2020); Jorgensen et al. (2020); Kuang et al. (2020); Rathish et al. (2021); Tumbarello et al. (2021); Zhu et al. (2021)	Combination therapy drug	Country	Study design	No. of patients	Sex, male [*n* (%)]	Age median (IQR)	Bacteria	Carbapenemase	CCI [median (IQR)]	The severity of illness [median (IQR)]	Infection type
King et al. (2017) ^a^	AMG, PM, and TGC	USA	Ret	60^b^	36 (60)	60 (51–69)	CRE	NR	4.5 (3–7)	2 (0–5)^c^	Mix
Temkin et al. (2017) ^a^	ERP, AMG, PM, TGC, and MEM	Europe and Australia	CS	38^d^	25 (65.8)	61 (47–67)	CRE and CRPA	KPC and OXA-48	NR	NR	Mix
Sousa et al. (2018) ^e^ ^,^ ^f^	COL, TGC, AMK, and CAP	Spain	Ret	57^g^	44 (77)	64 (range, 26–86)	CPE	OXA-48	3 (0–13)	24 (8–45)^h^	Mix
De la Calle et al. (2019) ^i^	AMK, TGC, and COL	Spain	Ret	24^d^	19 (82.6)	58.85 (SD = 16.03)	CPE and *E. coli*	OXA-48	4.3 (SD = 2.9)	3.3 (SD = 2.8)^j^	Mix
Shields et al. (2018)	GEN, COL, TGC, AMK, and CIP	USA	Ret	77^d^	47 (61)	62 (range, 19–91)	CRE	KPC-2 and KPC-3	4 (range, 0–10)	5 (range, 0–20)^j^	Mix
Guimarães et al. (2019) ^e^	TGC, PM, MEM, AMK, GEN, and FOS	Brazil	CS	29^g^	18 (62)	50.5 (SD = 5)	CRE	KPC-2	2 (range, 0–5)	2 (range, 0–8)^c^	Mix
Tumbarello et al. (2019) ^f^	GEN, TGC, COL, FOS, and CAP	Italy	CC	104^d^	68 (65.4)	61 (27–79)	CKP	KPC	CCI ≥3 (36.5%)	4 (0–7)^c^	BSI
Iannaccone et al. (2020) ^a^	TGC, COL, GEN, FOS, and MEM	Italy	Cs	23^k^	21 (91)	57 (range, 18–80)	CKP	KPC	NR	NR	BSI
Jorgensen et al. (2020) ^f^	AMG, TGC, and PM	USA	Ret	109^d^	58 (53)	63 (53–74)	CRE	NR	4 (2–7)	5 (3–8)^j^	Mix
Castón et al. (2020) ^f^	GEN, TGC, COL, and FOS	Spain	Ret	47^d^	29 (61.7)	70 (54–79)	CKP	KPC	4 (2–6)	3 (2–6)^j^	Mix
Jiang et al. (2020)	CAP, AMK, TGC, and PM	China	Cs	41^d^	34 (82.9)	62.4 (SD = 15.2)	CKP	NR	NR	NR	Pneumonia
Chen et al. (2020) ^i^	TGC and PM	China	Ret	10^d^	10 (100)	51 (range, 31–68)	CKP and CRPA	KPC-2	NR	5.8 (range, 1–13)^j^	Mix
Ackley et al. (2020) ^i^	CAP, AMG, PM, COL, TGC, FLU, and SUL-TRI	USA	Ret	105^d^	58 (55.2)	62 (51–69)	CRE	Partial KPC	5 (3–6)	26 (22–30)^h^	Mix
Kuang et al. (2020) ^e^ ^,^ ^f^	TGC, PM, AMK, FOS, and MEM	China	Ret	20^d^	14 (70)	54.5 (SD = 17.37)	CRE, AB, and CRPA	NR	4.2 (SD = 2.46)	12.15 (SD= 4.63)^h^	Mix
Tumbarello et al. (2021) ^f^	FOS, TGC, GEN, MEM, COL, and AMK	Italy	Ret	577^d^	386 (66.9)	66 (56–76)	CKP	KPC	CCI ≥ 3 (84.7%)	INCREMENT score ≥8 (31.2%)	Mix
Zhu et al. (2021)	CAP, TGC, PM, and FLU	China	Cs	11^d^	7 (63.6)	55 (range, 38–69)	CKP	NR	NR	NR	Mix
Rathish et al. (2021) ^a^	TGC, PM, MEM, MIN, and FLU	India	Ret	103^g^	81 (79)	53.2 (SD = 17.3)	CRE	NR	4.1 (SD = 2.3)	4.3 (SD = 3.2)^j^	Mix

AB, *Acinetobacter baumannii*; AMG, aminoglycosides; AMK, amikacin; BSI, bloodstream infection; CAP, carbapenem; CC, case cohort; CCI, Charlson Comorbidity Index; CIP, ciprofloxacin; CKP, carbapenem-resistant *Klebsiella Pneumonia*; COL, colistin; CPE, carbapenemase-producing Enterobacteriaceae, CRE, carbapenem-resistant Enterobacteriaceae; CRPA, carbapenem-resistant *Pseudomonas aeruginosa*; CS, case series; Cs, case sectional; ERP, ertapenem; FLU, fluoroquinolone; FOS, fosfomycin; GEN, gentamicin; IMP, imipenem; IQR, interquartile range; MEM, meropenem; MIN, minocycline; NR, not report; PM, polymyxin; Ret, retrospective cohort; SD, standard deviation; SOFA, Sequential Organ Failure Assessment; SUL-TRI, sulfamethoxazole-trimethoprim; TGC, tigecycline.

a In-hospital mortality.

b Minimum therapy time > 24 h.

c Pitt score.

d Minimum therapy time > 72 h.

e 14-day mortality.

f 30-day mortality.

g Minimum therapy time > 48 h.

h APACHE Ⅱ score.

i 90-day mortality.

j SOFA score.

k Not report minimum therapy time.

**FIGURE 2:**
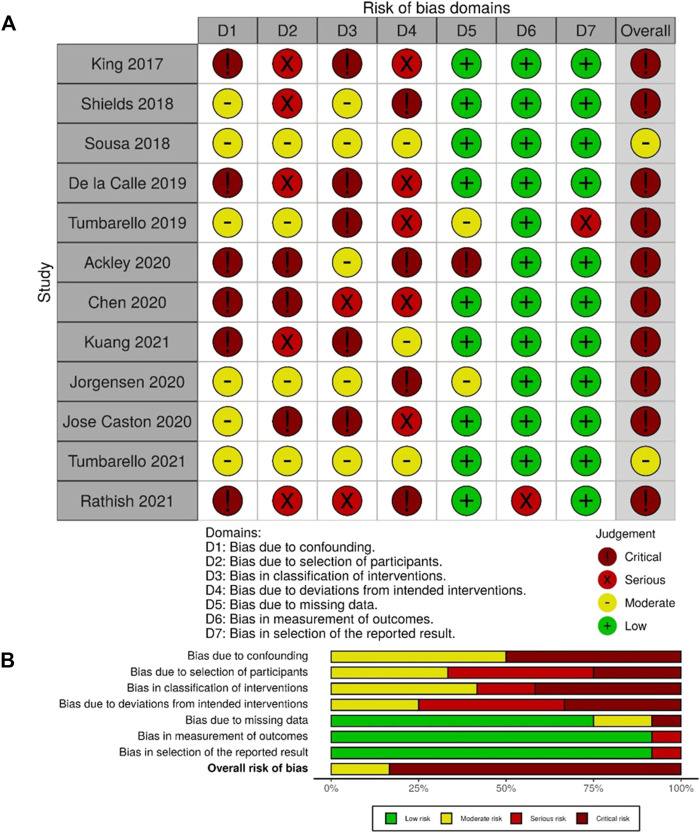
Risk of bias for each domain and each study in seven domains.

Robvis tool (McGuinness and Higgins, 2021) was applied for the publication quality (risk-of-bias) assessment. The risk-of-bias assessment results for each study (Figure 2A) and each domain (Figure 2B) are displayed. Ten studies (King et al., 2017; Shields et al., 2018; De la Calle et al., 2019; Tumbarello et al., 2019; Ackley et al., 2020; Castón et al., 2020; Chen et al., 2020; Jorgensen et al., 2020; Kuang et al., 2020; Rathish et al., 2021) have a critical overall risk of bias (judged by one study was at critical risk of bias in at least one domain (Sterne et al., 2016b)) and two moderates (Sousa et al., 2018; Tumbarello et al., 2021). The quality of the other five studies assessed by the AHRQ tool is all poor quality (Table 2).

**TABLE 2:** Quality assessment of five studies by AHRQ.

Study	1) Define the source of information (survey and record review)	2) List inclusion and exclusion criteria for exposed and unexposed subjects (cases and controls) or refer to previous publications	3) Indicate time period used for identifying patients	4) Indicate whether subjects were consecutive or not if not population-based	5) Indicate if evaluators of subjective components of the study were masked to other aspects of the status of the participants	6) Describe any assessments undertaken for quality assurance purposes (e.g., test/retest of primary outcome measurements)	7) Explain any patient exclusions from analysis	8) Describe how confounding was assessed and/or controlled	9) If applicable, explain how missing data were handled in the analysis	10) Summarize patient response rates and completeness of data collection	11) Clarify what follow-up, if any, was expected and the percentage of patients for which incomplete data or follow-up was obtained	The overall quality
Iannaccone et al. (2020)	Yes	No	Yes	Yes	Yes	No	No	N0	No	No	No	low
Temkin et al. (2017)	Yes	No	No	Unclear	Yes	No	No	No	No	No	No	low
Guimaraes et al. (2019)	Yes	Yes	Yes	Yes	Yes	No	No	No	No	No	No	low
Zhu et al. (2021)	Yes	Yes	Yes	Unclear	Yes	No	No	No	No	No	No	low
Jiang et al. (2020)	Yes	Yes	Yes	Unclear	Yes	No	No	No	No	No	No	low

### Meta-Analysis

#### Mortality

**FIGURE 3:**
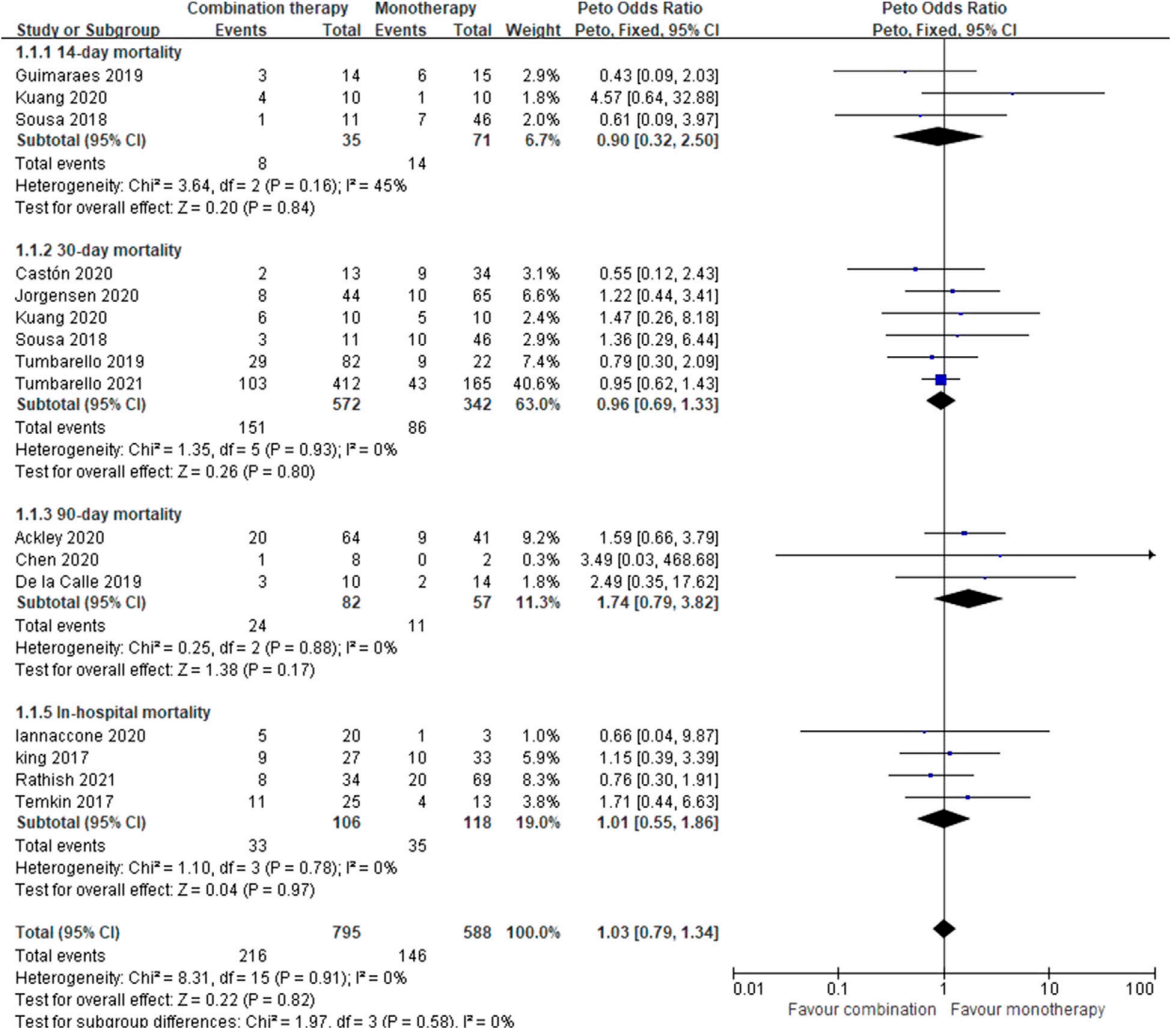
Mortality of CAZ-AVI therapy alone versus in combination.

Out of the 17 included eligible studies, three studies (Shields et al., 2018; Jiang et al., 2020; Zhu et al., 2021) did not extract separate data on mortality rates from CAZ-AVI therapy alone or in combination with other drugs. Given the fact that most studies’ overall risk of bias was critical, a subgroup meta-analysis was not performed based on the risk-of-bias levels but on mortality at an appropriate time point (14, 30, and 90 days, in hospital). Sensitivity analyses were performed according to the study design, comorbidity index, illness severity, bacteria, infection type, and carbapenemase. There was no statistically significant difference in the overall mortality rates between CAZ-AVI therapy alone and ceftazidime-avibactam-based combination therapy (Peto OR = 1.03, 95% CI 0.79–1.34; *I* ^2^ = 0%) (Figure 3).

For the 14-day mortality, the Peto OR was 0.90 (95% CI 0.32–2.50; *I* ^2^ = 45%) with no significant difference, but there were only three articles (Sousa et al., 2018; Kuang et al., 2020; Guimarães et al., 2019) and 106 patients included. Moderate clinical heterogeneity (*I* ^2^ = 45%; *p* = 0.16) was observed for one report (Kuang et al., 2020), which may be due to the pathogen complexity (including CRE, CRPA, and AB). By excluding this study, *I* ^2^ was 0%, and the Peto OR was 0.50 (95% CI 0.15–1.64). Six studies (Sousa et al., 2018; Kuang et al., 2020; Tumbarello et al., 2019; Castón et al., 2020; Tumbarello et al., 2021; Jorgensen et al., 2020) were used to assess the 30-day mortality rate, contributing to 63.0% of the weight. No significant heterogeneity existed among these studies (*I* ^2^ = 0%; *p* = 0.93), and there was no statistically significant difference between CAZ-AVI therapy and combination therapy (Peto OR = 0.96, 95% CI 0.69–1.33; *I* ^2^ = 0%). The Peto OR of 90-day mortality assessment was 1.74 (95% CI 0.79–3.82; *I* ^2^ = 0%), thus exhibiting no significant difference. Three studies (De la Calle et al., 2019; Ackley et al., 2020; Chen et al., 2020) consisting of 139 patients were included in this analysis. For the in-hospital mortality assessment, four studies (King et al., 2017; Temkin et al., 2017; Iannaccone et al., 2020; Rathish et al., 2021) were included with Peto OR = 1.01 (95% CI 0.55–1.86; *I* ^2^ = 0%).

#### Clinical Success

**FIGURE 4:**
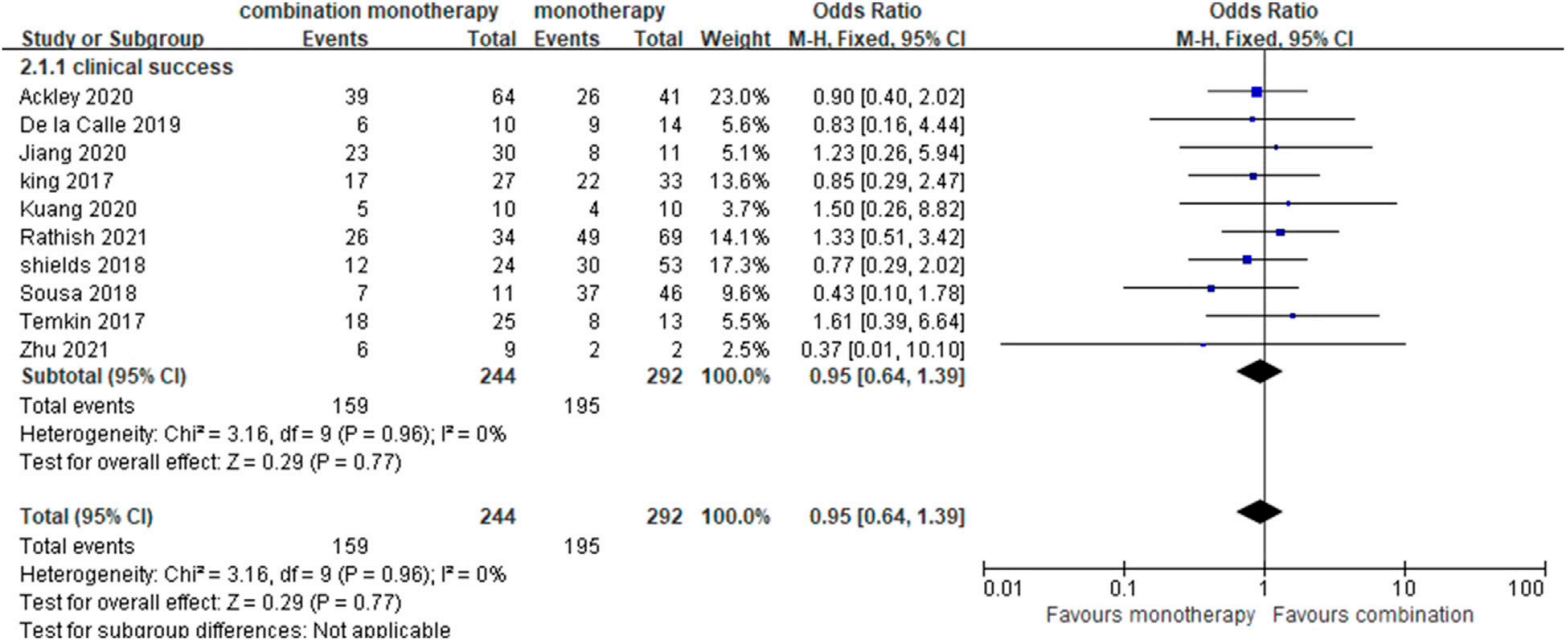
Clinical success of CAZ-AVI therapy alone versus in combination.

Ten studies (King et al., 2017; Temkin et al., 2017; Shields et al., 2018; Sousa et al., 2018; De la Calle et al., 2019; Ackley et al., 2020; Jiang et al., 2020; Kuang et al., 2020; Rathish et al., 2021; Zhu et al., 2021) were included in the clinical success analysis, in which only one (case series) study (Temkin et al., 2017) did not define clinical success, and the other nine studies defined clinical success as resolution or remarkable improvement of the symptoms and different signs of infection at some point (such as the endpoint) (Sousa et al., 2018; De la Calle et al., 2019; Ackley et al., 2020), absence of recurrent infections (Shields et al., 2018; De la Calle et al., 2019; Ackley et al., 2020), or resistance to CAZ-AVI (Rathish et al., 2021). Sensitivity analyses were performed according to the study design, definitions of clinical success, illness severity, and carbapenemase. However, no statistical difference was observed based on a pooled OR of 0.95 (95% CI 0.64–1.39; *I* ^2^ = 0%) (Figure 4).

#### Microbiologically Negative

**FIGURE 5:**
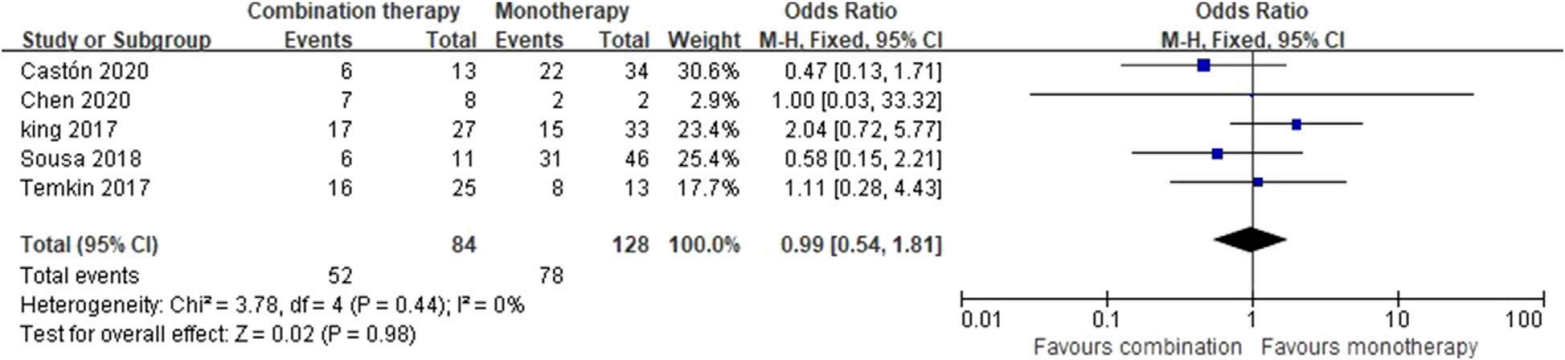
Microbiologically negative of CAZ-AVI therapy alone versus in combination.

Five studies (King et al., 2017; Temkin et al., 2017; Sousa et al., 2018; Castón et al., 2020; Chen et al., 2020) reported the outcomes of microbiologically negative, including a total of 212 patients. Out of these studies, three studies defined the microbiologically negative as sterilization of the index culture at the end of treatment or an appropriate point (7 days) (King et al., 2017; Sousa et al., 2018; Castón et al., 2020), while the other two studies had no definitions (Temkin et al., 2017; Chen et al., 2020). Sensitivity analyses were performed according to the study design, definitions of microbiologically negative, illness severity, and carbapenemase, and there was no significant difference. The pooled OR of the five studies was 0.99 (95% CI 0.54–1.81; *I* ^2^ = 0%) (Figure 5).

#### Posttreatment Resistance of CAZ-AVI

**FIGURE 6:**
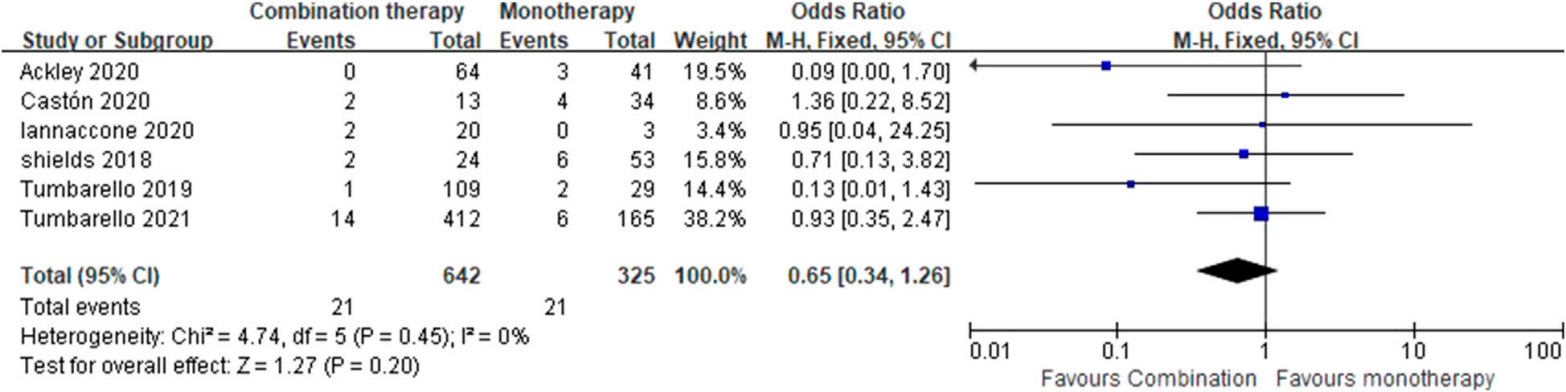
Posttreatment resistance of CAZ-AVI therapy alone versus in combination.

Six studies (Shields et al., 2018; Tumbarello et al., 2019; Ackley et al., 2020; Iannaccone et al., 2020; Castón et al., 2020; Tumbarello et al., 2021) assessed the development of resistance after CAZ-AVI treatment, in which 967 patients (642 in CAZ-AVI-based combination therapy and 325 in CAZ-AVI therapy) were included. Sensitivity analyses were performed by the study design, the illness severity, comorbidity index, and infection type. Heterogeneity was either low or absent, and no difference was observed (Figure 6). *Klebsiella pneumoniae* carbapenemase- (KPC-) producing isolates were reported in all the six studies, with one study (Shields et al., 2018) reporting both KPC-2 and KPC-3 and another study (Ackley et al., 2020) reporting parts of the isolates as KPC-producing bacteria. In our analysis, there was no significant difference (OR = 0.65, 95% CI 0.34–1.26; *I* ^2^ = 0%). However, when we pooled only three studies (Tumbarello et al., 2019; Ackley et al., 2020; Iannaccone et al., 2020) including 266 participants, the pooled OR was 0.18 (95% CI 0.04–0.78; *I* ^2^ = 0%) favoring CAZ-AVI-based combination therapy. Therefore, we observed a posttreatment resistance trend in CAZ-AVI alone therapy (Figure 6 **,** *p*-value = 0.2). Further study with larger samples would need to be conducted to confirm this assumption.

## Discussion

The mortality, clinical success, microbiologically negative, and posttreatment resistance between CAZ-AVI therapy alone and in combination with other agents were compared. The results indicate that CAZ-AVI concomitant therapy may not exhibit beneficial effects on mortality, clinical success, and microbiologically negative to patients infected with CRGN. This is consistent with the two previous meta-analyses (one reported only mortality (Fiore et al., 2020), and the other one reported mortality and microbiologically negative (Onorato et al., 2019)). However, a trend of posttreatment resistance is likely to occur in the CAZ-AVI therapy based on the pooled three studies, OR = 0.18, 95% CI 0.04–0.78).

Four subgroups were analyzed on data regarding mortality, and the discrepancies were not significant, which is consistent with previous studies reported by Fiore (Fiore et al., 2020) and Onorato (Onorato et al., 2019). Gutiérrez-Gutiérrez et al. (Gutiérrez-Gutiérrez et al., 2017) reported no difference observed in overall mortality rates between patients who received antibiotics concurrent with other agents (colistin plus tigecycline most common) and monotherapy (any but not CAZ-AVI) treating carbapenemase-producing Enterobacteriaceae (CPE) infections. However, in the high-mortality-score patients, combination therapy was related to lower mortality compared with monotherapy. Another study conducted by Tumbarello (Tumbarello et al., 2015) demonstrated that combination therapy including meropenem improved survival rates in severity illness patients with KPC-Kp infections. Here, we performed sensitivity analyses according to the severity of illness, and no significant difference was observed.

Clinical success, which was first evaluated in our meta-analysis, showed no difference in the 10 studies (Sousa et al., 2018; Kuang et al., 2020; Rathish et al., 2021; Shields et al., 2018; Ackley et al., 2020; Zhu et al., 2021; King et al., 2017; Temkin et al., 2017; De la Calle et al., 2019; Jiang et al., 2020) included (OR = 0.95, 95% CI 0.64–1.39; *I* ^2^ = 0%). Results of microbiologically negative were evaluated in five studies (Sousa et al., 2018; Castón et al., 2020; King et al., 2017; Temkin et al., 2017; Chen et al., 2020) and showed no discrepancies (OR = 0.99, 95% CI 0.54–1.81; *I* ^2^ = 0%). Out of the five studies, two studies (Temkin et al., 2017; Chen et al., 2020) did not define the microbiologically negative. Besides, to the best of our knowledge, this was the first meta-analysis to systematically evaluate the development of resistance after CAZ-AVI treatment alone or in combination with other agents. CAZ-AVI was first approved by the US FDA (Tumbarello et al., 2019) in 2015, and subsequently, rapidly emerging resistance was first reported by Humphries et al. (Humphries et al., 2015). In their study, an old woman with infections caused by KPC-producing KP (KPC-3) received salvage therapy and developed resistance to CAZ-AVI after 9 days of treatment (Humphries et al., 2015). In our meta-analysis, six studies (Shields et al., 2018; Tumbarello et al., 2019; Ackley et al., 2020; Iannaccone et al., 2020; Castón et al., 2020; Tumbarello et al., 2021) reported posttreatment resistance of CAZ-AVI, and only one study (Shields et al., 2018) provided detailed information on the potential mechanism of CAZ-AVI resistance that both KPC-2- and KPC-3-producing CRE were included (Shields et al., 2018). Interestingly, resistance emerged only from KPC-3-producing isolates. This is consistent with the study reported by Humphries et al. (Humphries et al., 2015). Further study is needed to unravel the potential resistance mechanism of CAZ-AVI posttreatment. Additionally, although there was no significant difference (OR = 0.65, 95% CI 0.34–1.26; *I* ^2^ = 0%) in CAZ-AVI resistance between treatment in combination and alone, combination therapy was more likely associated with lower resistance when we pooled three studies (Tumbarello et al., 2019; Ackley et al., 2020; Iannaccone et al., 2020) (OR = 0.18, 95% CI 0.04–0.78; *I* ^2^ = 0%). Further studies with a larger sample size are needed to test this assumption. Given that CAZ-AVI concomitant with other antibiotics (such as TGC or COL) may result in potential side effects or toxicity (Sousa et al., 2018), decisions regarding CAZ-AVI-based combination therapy should be made according to the traits of patients.

This study has several limitations. Firstly, all studies included were retrospective observational design studies, with no RCTs or prospective observational studies included. Secondly, the overall risk of bias of most studies was critical. Thirdly, the sample sizes of several subgroups were small. Fourth, due to the fact that the data on the type of infection between CAZ-AVI therapy alone or in combination with other agents were not available, subgroup analysis was not performed according to the infection type.

In summary, studies for CAZ-AVI single therapy or CAZ-AVI combination therapy in patients with CRGN infections were analyzed. The outcomes including mortality, clinical success, microbiologically negative, and posttreatment resistance were compared, and no significant differences were observed between the two therapies. To the best of the authors’ knowledge, this is the first meta-analysis study accessing the resistance between CAZ-AVI treatment alone and in combination. However, given the low quality of the evidence and limited samples in some subgroups, definitive conclusions cannot be made. RCTs and large prospective observational studies are needed to evaluate these issues in future studies.

## Data Availability

The original contributions presented in the study are included in the article/Supplementary Material; further inquiries can be directed to the corresponding authors.
