# Supplementary material for: Ceftazidime-Avibactam Therapy Versus Ceftazidime-Avibactam-Based Combination Therapy in Patients With Carbapenem-Resistant Gram-Negative Pathogens: A Meta-Analysis
Source: Front Pharmacol. 2021 Sep 14;12:707499. doi: 10.3389/fphar.2021.707499 (PMC8476997; doi:10.3389/fphar.2021.707499)
Supplement: Supplementary file 2 [file DataSheet1.docx]

Search strategy as supplementary data

Search strategy:

To maximize the number of papers found, both medical subject headings and free text terms were used in the search strategy. The first search was performed until 30th January 2021. We updated searches to 31th March 2021. Multiple databases, including PubMed, EMbase, Web of Science, CNKI, and Wanfang Data, were searched for the studies on CAZ-AVI therapy and CAZ-AVI-based combination therapy for the treatment of infections caused by CRGN.

For PubMed we used the advanced search interface, and used the search string (‘ceftazidime avibactam’) and (‘carbapenem resistant’) to run searches.

For Embase we used the advanced search interface, and used the search string (‘ceftazidime avibactam’) and (‘carbapenem resistant’) to run searches.

For Web of Science we used the advanced search interface, and used the search string TS= (‘ceftazidime avibactam’ and ‘carbapenem resistant’) to run searches.

For CNKI we used the advanced search interface, and used the search string (‘ceftazidime avibactam’) and (‘carbapenem resistant’) to run searches.

For Wanfang database we used the advanced search interface, and used the search string (‘ceftazidime avibactam’) and (‘carbapenem resistant’) to run searches.

The references of relevant articles (both original research and review) were also inspected. The article search and reference inspection were performed by two researchers independently. In total, the search yielded 1106 records. After de-duplication, 667 unique records remained.
